# Modelling local structural and electronic consequences of proton and hydrogen-atom uptake in VO_2_ with polyoxovanadate clusters†

**DOI:** 10.1039/d1sc02809j

**Published:** 2021-08-25

**Authors:** Sourav Chakraborty, Eric Schreiber, Karla R. Sanchez-Lievanos, Mehrin Tariq, William W. Brennessel, Kathryn E. Knowles, Ellen M. Matson

**Affiliations:** Department of Chemistry, University of Rochester Rochester NY 14627 USA matson@chem.rochester.edu

## Abstract

We report the synthesis and characterisation of a series of siloxide-functionalised polyoxovanadate–alkoxide (POV–alkoxide) clusters, [V_6_O_6_(OSiMe_3_)(OMe)_12_]^*n*^ (*n* = 1−, 2−), that serve as molecular models for proton and hydrogen-atom uptake in vanadium dioxide, respectively. Installation of a siloxide moiety on the surface of the Lindqvist core was accomplished *via* addition of trimethylsilyl trifluoromethylsulfonate to the fully-oxygenated cluster [V_6_O_7_(OMe)_12_]^2−^. Characterisation of [V_6_O_6_(OSiMe_3_)(OMe)_12_]^1−^ by X-ray photoelectron spectroscopy reveals that the incorporation of the siloxide group does not result in charge separation within the hexavanadate assembly, an observation that contrasts directly with the behavior of clusters bearing substitutional dopants. The reduced assembly, [V_6_O_6_(OSiMe_3_)(OMe)_12_]^2−^, provides an isoelectronic model for H-doped VO_2_, with a vanadium(iii) ion embedded within the cluster core. Notably, structural analysis of [V_6_O_6_(OSiMe_3_)(OMe)_12_]^2−^ reveals bond perturbations at the siloxide-functionalised vanadium centre that resemble those invoked upon H-atom uptake in VO_2_ through *ab initio* calculations. Our results offer atomically precise insight into the local structural and electronic consequences of the installation of hydrogen-atom-like dopants in VO_2_, and challenge current perspectives of the operative mechanism of electron–proton co-doping in these materials.

## Abstract 2

We report the synthesis and characterisation of a series of siloxide-functionalised polyoxovanadate–alkoxide clusters, [V_6_O_6_(OSiMe_3_)(OMe)_12_]^*n*^ (*n* = 1^−^, 2^−^), that serve as molecular models for proton and hydrogen-atom uptake in vanadium dioxide.

## Graphical abstract

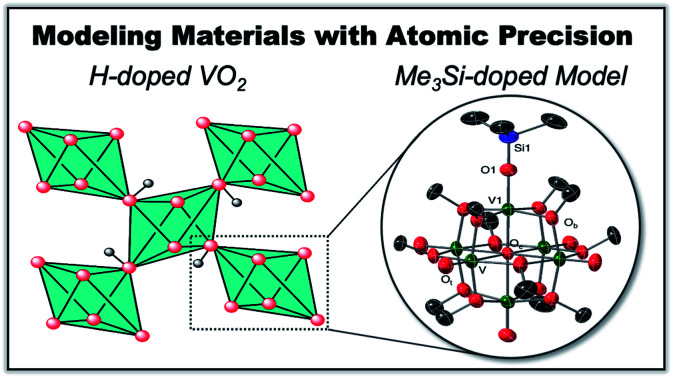

## Introduction

**Fig. 1:**
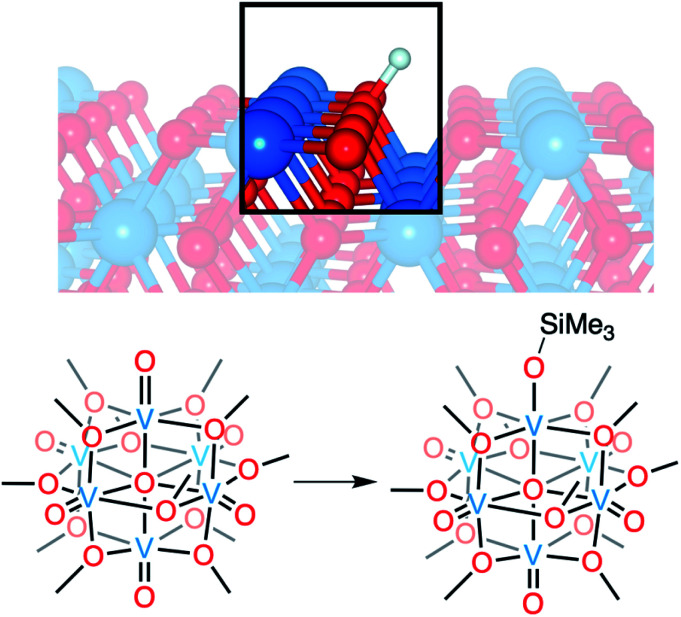
Modelling proton and hydrogen-atom uptake in VO_2_ (top) using POV–alkoxide clusters (bottom). Silylium cations are used as surrogates for protons to improve cluster stability and facilitate crystallographic resolution.

Vanadium dioxide (VO_2_) is a versatile material that can access a variety of crystal phases, each with its own chemical, electronic, and physical properties.^1^ These properties can be further tuned through the incorporation of impurities/dopants, improving the performance of VO_2_ in energy-saving smart window technologies and post lithium-ion battery cathode materials.^1–6^ In particular, the incorporation of hydrogen atoms (H-atoms) as dopants at exterior/surface sites of VO_2_ has been shown to tune the material's optoelectronic properties by stabilizing the conductive rutile phase of the material (VO_2_(R); Fig. 1).^7–11^ H-atom equivalents can be introduced to monoclinic VO_2_ (VO_2_(M)) post-synthetically *via* hydrothermal,^12^ hydrogen spillover,^13,14^ and most recently, electron–proton co-doping techniques.

Electron–proton co-doping of VO_2_(M) takes advantage of acidic protons and an applied voltage as the source of H-atoms, and is proposed to occur through the initial injection of an electron, followed by protonation of the lattice.^8,15^ Mechanistic analysis is complicated by poor resolution of structural perturbations that occur upon hydrogen uptake;^8,13,15^ the small size of the hydrogen nucleus precludes definitive resolution of changes to the proximal lattice structure of the material. As such, insight into the mechanism of H-atom uptake is largely based on computational modelling, which does not explicitly take into account the role that the coupled uptake of protons and electrons might play in lowering the kinetic barrier of the reduction of this insulating material at room temperature. Indeed, recent work from Mayer and coworkers has demonstrated that electrochemical H-doping of metal oxides proceeds *via* proton-coupled electron transfer, whereby the presence of acidic protons in the electrolyte facilitate reduction.^16^ These results call the original proposal for the post synthetic formation of H-doped VO_2_ into question.

One popular approach that offers atomic-level insight into the mechanism and local electronic consequences of hydrogen uptake involves the use of molecular metal oxide cluster complexes as models for bulk solids.^17–20^ Unlike the majority of POVs reported that are isolated with high-valent vanadium(v) centres,^21–23^ Lindqvist-type polyoxovanadate–alkoxide (POV–alkoxide) clusters can be isolated in a reduced state, where all six vanadium ions adopt tetravalent oxidation states, allowing our group to study these compounds as models for VO_2_.^24–26^ Additionally, the vanadium centres within these clusters adopt edge-sharing pseudo-octahedral coordination geometries that are analogous to the immediate chemical environment of vanadium(iv) ions in all known phases of VO_2_.^27–30^ While these clusters possess delocalised electronic structures that can mimic local electronic communication between adjacent vanadium centres, they lack the extended band structure and Mott physics of the bulk material, limiting the effectiveness of our model to understanding the local consequences of surface reactivity and atomistic changes within VO_2_.

Previously, we reported the reactivity of a reduced POV–alkoxide cluster with protons in an attempt to access models of proton uptake in VO_2_.^31^ Upon addition of an organic acid to the reduced cluster, [^*n*^Bu_4_N]_2_[V^IV^_6_O_7_(OEt)_12_] (Et = C_2_H_5_), formation of an oxygen-deficient assembly was observed. The proposed mechanism invokes the formation of a transient hydroxide-functionalised species, [V_6_O_6_(OH)(OEt)_12_]^1−^, which undergoes rapid intermolecular disproportionation. The hydroxide functionalised assembly is intriguing, as its isolation would provide a handle to interrogate the structural and electronic consequences, as well as the mechanism of H-doping in VO_2_ in acidic medium.

For decades, trialkylsilylium cations have been used as surrogates for protons in organic synthesis. Researchers have noted that silylium ions are capable of mediating analogous chemical transformations to protons, specifically in examples involving the reduction of carbon–oxygen multiple bonds.^32–34^ More recently, this analogy has been extended to the use of organosilanes as hydrogen-like reagents in inorganic syntheses.^35,36^ Drawing inspiration from these works, we hypothesised that the use of SiR_3_^+^ as a bulky surrogate for a proton would improve the stability of the siloxide-equivalent of the unobserved hydroxide intermediate, thus allowing for its isolation and analysis.

Here, we present the synthesis and characterisation of a siloxide-functionalised POV–alkoxide cluster, [V^IV^_6_O_6_(OSiMe_3_)(OMe)_12_]^1−^ (Me = CH_3_), generated *via* addition of trimethylsilyl trifluoromethylsulfonate (TMSOTf) to [V^IV^_6_O_7_(OMe)_12_]^2−^ (**1-V6IVO72−**; Fig. 1). Notably, binding of the silylium ion to a surface vanadyl site enables reduction of the cluster by a single electron. The added reducing equivalent introduces a site-differentiated V^III^ ion into the Lindqvist core adjacent to the silylium dopant. This result suggests that cation coordination to the surface of the vanadium(iv) oxide assembly is required for reduction, challenging the established sequential addition of electrons then protons believed to be necessary to access H-doped VO_2_. Collectively, this work provides atomically precise insights into the local structural and electronic impacts of H-atom dopants in VO_2_, as well as mechanistic inferences that suggest proton-coupled electron transfer may be a valid alternative pathway for H-atom uptake in VO_2_.

## Results and discussion

**Scheme 1:**
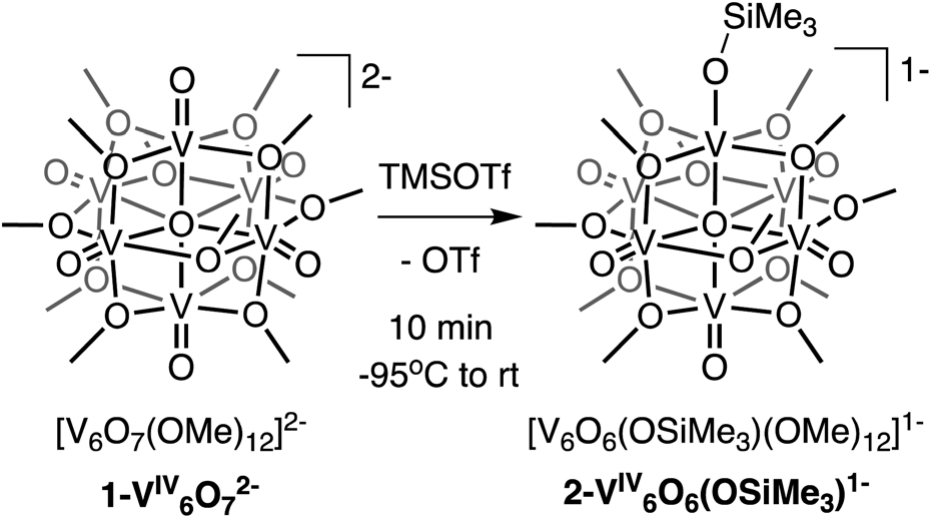
Synthesis of complex **2-V6IVO6(OSiMe3)1−**.

We first investigated the reactivity of TMSOTf with **1-V6IVO72−**. We opted to use the methoxide-bridged variant of the POV–alkoxide cluster to minimise steric clashes between surface ligands and the trimethylsilylium ion. Addition of one equivalent of TMSOTf to a bright blue solution of complex **1-V6IVO72−** at low temperature resulted in an immediate colour change to green (Scheme 1). Following workup, analysis of the reaction mixture by ^1^H NMR spectroscopy revealed three new paramagnetically shifted and broadened resonances (*δ* = 27.50, 24.12 and −9.79 ppm, Fig. S1†). This pattern is consistent with the expected reduction of symmetry of the parent cluster (pseudo-*O*_h_ → pseudo-*C*_4v_) that would accompany surface functionalisation.^37–39^ Additional characterisation of the product by electrospray ionization mass spectrometry (ESI-MS, (−) mode) revealed a peak located at *m*/*z* = 863, corresponding to a molecular weight consistent with the addition of a single trimethylsilyl moiety to complex **1-V6IVO72−** (Fig. S2†). Collectively, these data suggest successful formation of the siloxide-functionalised assembly, **2-V6IVO6(OSiMe3)1−**.

**Fig. 2:**
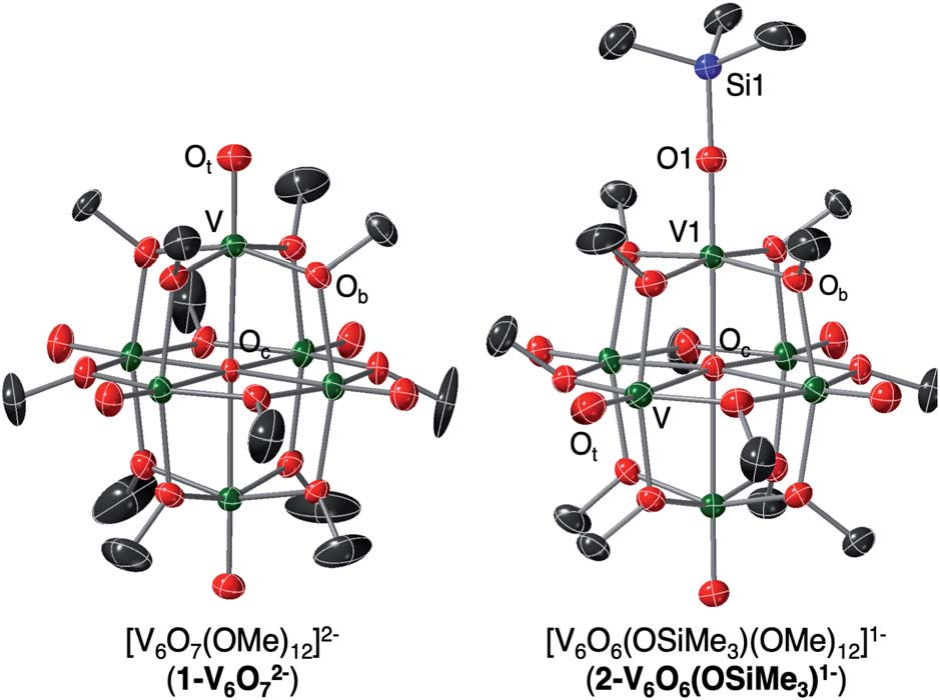
Crystal structures of **1-V6IVO72−** and **2-V6IVO6(OSiMe3)1−** shown with 30% probability ellipsoids. Hydrogen atoms, counter ions, and solvent molecules have been removed for clarity.

To unambiguously identify the molecular structure of **2-V6IVO6(OSiMe3)1−**, crystals suitable for analysis *via* single crystal X-ray diffraction (SCXRD) were grown from slow diffusion of diethyl ether into a concentrated solution of the product in dichloroethane (Fig. 2 and Table 1, see Table S1† for complete crystallographic data and refinement parameters). Refinement of structural data confirms installation of a trimethylsilyl cation at a single vanadyl moiety. Broadly speaking, bond metrics of the Lindqvist ion remain constant following attachment of the silyl moiety to the surface of the assembly. The only exception to this observation is the V1–O bond lengths of the site differentiated vanadium centre. Indeed, activation of the vanadyl moiety *via* formation of the siloxide ligand manifests in a significant reduction of the V1–O1 bond order, as indicated by the elongation of the V1–O1 bond (V1–O1 (**2-V6IVO6(OSiMe3)1−**) = 1.768(3) Å *vs.* V=O_t_ (avg, **1-V6IVO72−**) = 1.606(1) Å; O_t_ = terminal oxido moiety). The V1–O1 bond length is reminiscent of previously reported mononuclear vanadium(iv) complexes bearing siloxide ligands (1.761–1.772 Å).^40,41^ Additionally, activation of the terminal oxido ligand following silylium coordination results in significant truncation of the V1–O_c_ bond (V1–O_c_ (**2-V6IVO6(OSiMe3)1−** = 2.019(3) Å *vs.* V–O_c_ (avg. **1-V6IVO72−**) = 2.311(6) Å; O_c_ = central μ_6_-oxido moiety). The V1–O_c_ bond distance resembles values reported previously upon defect formation in POV–alkoxide clusters (2.0666(14)–2.120(5) Å).^37–39^

**Table 1:** Selected bond lengths and angles of complexes **1-V6IVO72−**, **2-V6IVO6(OSiMe3)1−**, and **3-VIIIV5IV(OSiMe3)2−**

Bond/angle	**1-V6IVO72−** a, e^−^ distrib. = V^IV^_6_	**2-V6IVO6(OSiMe3)1−**, e^−^ distrib. = V^IV^_6_	**3-VIIIV5IVO6(OSiMe3)2−**, e^−^ distrib. = V^III^V^IV^_5_
V*n*–O_t_ (avg.)	1.606(1) Å (*n* = 1–3)	1.595(6) Å (*n* = 2–6)	1.6010(4) Å (*n* = 2–6)
V*n*–O_c_ (avg.)	2.311(6) Å (*n* = 1–3)	2.342(33) Å (*n* = 2–5)	2.323(30) Å (*n* = 2–5)
V1–O1	—	1.768(3) Å	1.929(4) Å
V1–O_c_	—	2.019(3) Å	2.138(3) Å
V1–O1–Si1	—	174.8(2)° (major, 78%)	176.3(3)° (major 78%)
		160.5(6)° (minor, 22%)	151.1(8)° (minor 22%)

a Bond distances obtained from previously reported structural analysis of complex **1-V6IVO72−**.^51^

Surface functionalisation of polyoxometalates (POMs) *via* the addition of silylium cations has been previously reported. However, most studies have been rooted in the development of methodologies for the organofunctionalisation of polyoxotungstates, focused on the formation of bridging siloxide ligands at the nucleophilic terminal oxide ligands of lacunary assemblies.^42–50^ By contrast, the formation of terminal siloxide moieties at the surface of plenary POMs, as observed following addition of TMSOTf to **1-V6IVO72−**, is quite rare. The single example is reported in the case of a niobium-functionalised polyoxotungstate ion, [Nb_2_W_4_O_19_]^4−^.^51^ In this work, addition of *tert*-butyldimethylsilyl triflouromethylsulfonate results in the silylation of a lone terminal Nb^V^=O bond to generate a siloxide ligand, similar to that observed in the case of complex **2-V6IVO6(OSiMe3)1−**. The authors credit the selectivity of this reaction for the terminal Nb=O ligand to the steric bulk of the selected silylium cation, noting that smaller electrophiles (*e.g.* protons, methyl cations, trimethylsilylium ions), bind preferentially to the more nucleophilic, bridging oxide positions. Here, we have eliminated the possibility of the engagement of μ_2_-oxido ligands by fully saturating these bridging positions with alkoxide moieties.

Crystallographic characterisation of **2-V6IVO6(OSiMe3)1−** provides atomic-level insight for local structural changes that occur upon proton uptake in VO_2_. Analysis of the site-differentiated vanadium ion within the POV–alkoxide cluster reveals a slight changes in V–O bond distances along the *z*-axis of V1, which, through ligand field analysis, would translate to the stabilization of the d_z^2^_ orbital of V1. This result is notable, as previous work has indicated that changes in the electronic properties of VO_2_ upon proton uptake occur as a result of the modification of the energetics of the d_z^2^_ orbital of the doped ions.^8,10,13,52^

### Implications of silylium coordination on the electronic structure of **1-V6IVO72−**

Toward the goal of using reduced POV–alkoxide clusters to model changes in the local electronic properties of VO_2_ upon proton uptake, we next analysed the electronic structure of complex **2-V6IVO6(OSiMe3)1−**. The siloxide-functionalised POV–alkoxide cluster crystallises with each vanadium ion in a unique position within the unit cell, enabling the assignment of the oxidation states of individual vanadium centres using bond valence sum (BVS) calculations (Table S2†).^53,54^ All vanadium centres within the hexavanadate assembly were identified as tetravalent ions, resulting in an overall oxidation state distribution of V^IV^_6_ for complex **2-V6IVO6(OSiMe3)1−**. This assignment is identical to that of the parent cluster, **1-V6IVO72−** (V^IV^_6_).

**Fig. 3:**
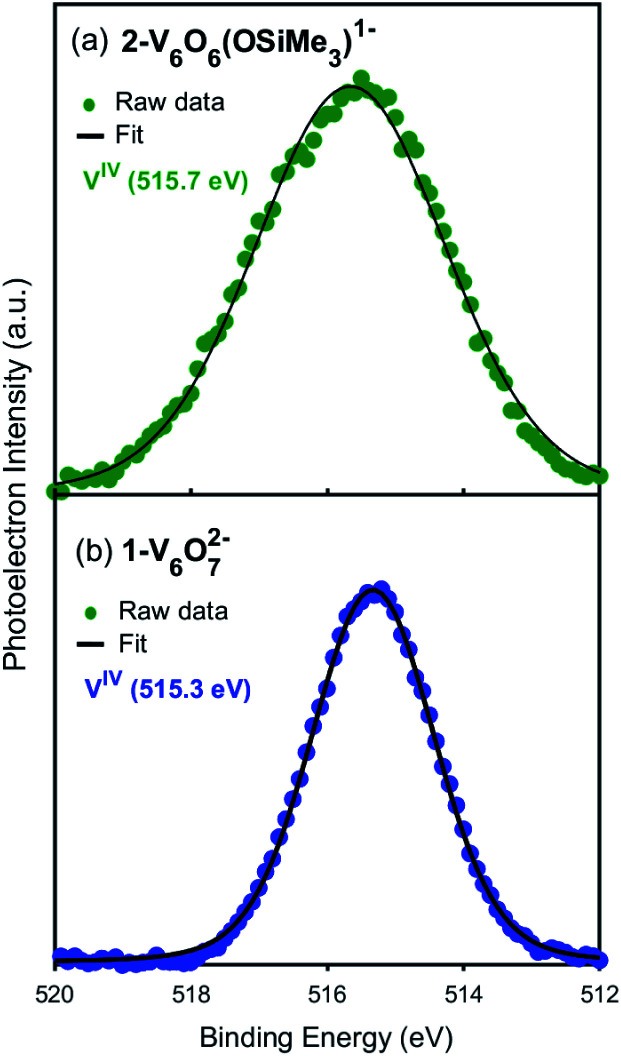
X-ray photoelectron spectra of the V 2p_3/2_ region of (a) **2-VIV6O6(OSiMe3)1−** and (b) **1-VIV6O72−**.

Additional evidence for the retention of the oxidation state distribution upon silylium coordination was obtained *via* X-ray photoelectron spectroscopy (XPS, Fig. 3). This analytical technique provides valuable information about the oxidation states and chemical environment of elements present in a solid sample by measuring the binding energies of photo-ejected core electrons. The XPS data of complex **1-V6IVO72−** exhibits a single V 2p_3/2_ peak with a binding energy of 515.3 eV, consistent with literature values for V^IV^.^55^ Similarly, in the case of complex **2-V6IVO6(OSiMe3)1−**, a single V 2p_3/2_ peak is observed with a binding energy of 515.7 eV, suggesting this Lindqvist ion also possesses a hexavanadate core comprised of solely tetravalent vanadium ions. In contrast, the mixed valent monoanionic parent cluster [V_6_O_7_(OMe)_12_]^1−^ exhibits two peaks in the V 2p_3/2_ region with binding energies of 515.7 eV and 517.5 eV that correspond to V^IV^ and V^V^, respectively (Fig. S3†). The assignment of oxidation states in these clusters was corroborated by comparison to their solid-state analogues (*i.e.* VO_2_); in vanadium oxides, the oxidation state of vanadium is estimated by the difference in binding energy between the O 1s and V 2p_3/2_ orbital levels.^55,56^ These energy differences in **1-V6IVO72−** and **2-V6IVO6(OSiMe3)1−** are 14.7 and 14.9 eV, respectively, which matches well with previous reports on VO_2_.^57^ It is worth noting that a small shift in binding energy is observed when comparing the spectra of complexes **1-V6IVO72−** and **2-V6IVO6(OSiMe3)1−**, which is likely due to the sensitivity of core electrons to changes in their local bonding environment.^58^ The coordination of a silylium ion to the surface of the cluster induces a small shift toward higher binding energy in **2-V6IVO6(OSiMe3)1−**, indicating that silylium coordination reduces the electron density around the vanadium metal centres across the entire assembly.

Comparison of the electronic structures of the siloxide functionalised POV–alkoxide cluster, **2-V6IVO6(OSiMe3)1−**, and its chloride-doped congener, [V^III^V^IV^_4_V^V^O_6_Cl(OEt)_12_]^−1^, allows for comparison of the local electronic environments surrounding surface-bound cationic and substitutional anionic dopants in VO_2_, respectively.^24^ Previously, we reported that substitution of a surface oxido ligand for a chloride moiety results in the redistribution of electron density across the cluster core; two adjacent V^IV^ ions disproportionate to form a high-valent vanadyl centre (V^V^=O) and a site differentiated V^III^–Cl moiety.^24^ The change in electronic structure upon formation of the chloride-doped assembly was confirmed by XPS; after fitting the data collected for [V^III^V^IV^_4_V^V^O_6_Cl(OEt)_12_]^1−^, an oxidation state distribution of V^III^V^IV^_4_V^V^ was confirmed. In contrast, spectroscopic analysis of complex **2-V6IVO6(OSiMe3)1−** reveals perturbations of the electronic structure of the fully-oxygenated parent cluster, **1-V6IVO72−**, but does not support the presence of either V^III^ or V^V^ centres in the functionalised assembly. Instead, both assemblies are found to possess an isovalent oxidation state distribution of vanadium ions within the Lindqvist assembly (*e.g.* V^IV^_6_).

With appropriate caution, consideration of the differences in the changes in the electronic structures of complexes **2-V6IVO6(OSiMe3)1−** and [V^III^V^IV^_4_V^V^O_6_Cl(OEt)_12_]^1−^, compared to that of their fully-oxygenated precursors, [V^IV^_6_O_7_(OR)_12_]^2−^, can provide insight into the consequences of the formation of surface-bound and substitutional dopants, respectively, in VO_2_. While both categories of dopants have been credited with injecting carrier density into the bulk material, it is clear from the local changes in electronic structure upon dopant formation in POV–alkoxide clusters that the mechanisms by which these changes in electronic structure take place are distinct. In the case of the chloride-doped assembly, formal reduction of the doped vanadium ion is observed, requiring redistribution of electron density across the cluster core.^24^ This results in the formation of a charge-separated species, where the chloride-functionalised vanadium centre is electronically decoupled from the remaining vanadyl ions of the assembly. In contrast, silylium coordination to the surface of the POV–alkoxide cluster reveals only small changes in the electronic structure of the low-valent vanadium oxide assembly; the Lindqvist cluster can tolerate the addition of the surface-bound dopant without major reorganization of electron density within the three-dimensional framework. This result suggests that changes in carrier density upon cation uptake in these materials are likely due to the change in reducibility of the lattice upon dopant incorporation. These considerations will be discussed in depth in connection with electrochemical analysis of **2-V6IVO6(OSiMe3)1−** (*vide infra*).

### Electrochemical consequences of silylium binding to **1-V6IVO72−**

**Fig. 4:**
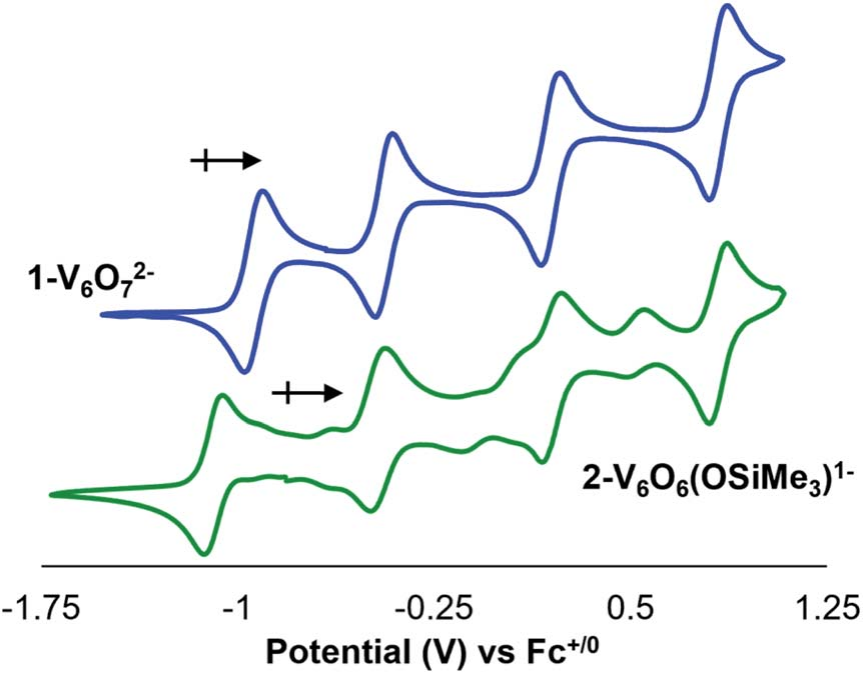
Cyclic voltammograms of complexes **1-VIV6O72−** (blue) and **2-VIV6O6(OSiMe3)1−** (green) collected in dichloromethane with 0.1 M [^*n*^Bu_4_N][PF_6_] as the supporting electrolyte (scan rate = 20 mV s^−1^). Note that impurities observed in the CV of **2-V6O6(OSiMe3)1−** result from oxidative instability of the siloxide-functionalised cluster.

To further interrogate changes in the electronic properties of the POV–alkoxide cluster upon silylium coordination, complex **2-V6IVO6(OSiMe3)1−** was characterised *via* cyclic voltammetry (CV). Analysis of the electrochemical profile of **2-V6IVO6(OSiMe3)1−** in dichloromethane reveals a voltammogram containing four quasi-reversible redox events (*E*_1/2_ = +0.84, +0.20, −0.47, −1.08 V *vs.* F^c+/0^; Fig. 4, Table 2). Comparing this to the CV of **1-V6IVO72−**, one notes striking similarities in the overall trace; the CV of the parent cluster likewise possesses four, one-electron redox events at potentials slightly shifted from that of **2-V6IVO6(OSiMe3)1−** (*E*_1/2_ = +0.84, +0.20, −0.44, −0.93 V *vs.* Fc^+/0^^59,60,63^ The open circuit potential (OCP) of complex **2-V6IVO6(OSiMe3)1−** was measured to be −0.78 V *vs.* Fc^+/0^, suggesting the isovalent V^IV^_6_ charge state lies between the two most reducing events. This observation indicates that silylium coordination stabilises an additional reduction event for the cluster core, corresponding to a V^IV/III^ process not observed in the parent assembly, **1-V6IVO72−**.

**Table 2:** Electrochemical parameters of complexes **1-V6IVO72−** and **2-V6IVO6(OSiMe3)1−** in dichloromethane

Redox couple	**1-V6O72−**	**2-V6O6(OSiMe3)1−**
	*E*_1/2_ a	*E*_1/2_ a
[V^IV^_2_V^V^_4_]/[V^IV^_3_V^V^_3_]	+0.84	—
[V^IV^_3_V^V^_3_]/[V^IV^_4_V^V^_2_]	+0.20	+0.84
[V^IV^_4_V^V^_2_]/[V^IV^_5_V^V^_1_]	−0.44	+0.20
[V^IV^_5_V_1_]/[V^IV^_6_]	−0.93	−0.47
[V^IV^_6_]/[V^III^_1_V^IV^_5_]	—	−1.08

a All *E*_1/2_ values are reported on the basis of data collected in dichloromethane and referenced *vs.* Fc^+/0^.

Reduction to V^III^ within a POV–alkoxide is a rare observation. Previous reports reveal that the formation of trivalent vanadium centres in cluster assemblies is possible upon formation of an oxygen defect; however, these ions typically remain electronically decoupled from other vanadyl sites in the core.^24,25,31,37–39^ Evidence for this phenomenon is observed upon chemical oxidation of the cluster; in all reported examples, oxidised forms of oxygen-deficient POV–alkoxide clusters retain a site-differentiated V^III^ centre at the defect site (*i.e.* the oxidation event occurs across vanadyl ions composing the remainder of the cluster core).^24,25,37,38^

Inspection of the CV of **2-V6IVO6(OSiMe3)1−** reveals its instability under electroanalytical conditions. Anodic scans of the cluster produce a mixture of species, as indicated by the appearance of new electrochemical features located at *E*_1/2_ = −0.704, −0.155, and +0.519 V *vs.* Fc^+/0^. The redox potentials of the “impurities” closely resemble values reported previously for the electrochemical profile of [V^III^V^IV^_5_O_6_(OMe)_12_]^1−^.^38^ The observed formation of the oxygen-deficient POV–alkoxide cluster is consistent with disproportionation of **2-V6IVO6(OSiMe3)1−**, resulting in an equimolar mixture of [V^III^V^IV^_5_O_6_(OMe)_12_]^1−^ and [V^IV^_5_V^V^O_7_(OMe)_12_]^1−^ and half an equivalent of hexamethyldisiloxane. This type of reactivity resembles that previously invoked for the putative hydroxide-functionalised POV–alkoxide cluster.^31^ Monitoring a dichloromethane solution of **2-V6IVO6(OSiMe3)1−***via*^1^H NMR spectroscopy confirms the suspected instability of the assembly; after 24 hours, full conversion of **2-V6IVO6(OSiMe3)1−** to a 1 : 1 ratio of [V^III^V^IV^_5_O_6_(OMe)_12_]^1−^ and [V^IV^_5_V^V^O_7_(OMe)_12_]^1−^ is observed (Fig. S4†). Notably, as one extends the range of the oxidative sweep past subsequent oxidation events, the current responses of redox processes corresponding to [V_6_O_6_(OMe)_12_]^*n*^ and [V_6_O_7_(OMe)_12_]^*n*^ increase in magnitude, which suggests that the concentrations of the products of disproportionation increase as the stability of the siloxide-functionalised cluster decreases under more oxidizing conditions (Fig. S5†).

### Modelling hydrogen-atom uptake in VO_2_(R); structural, electronic, and mechanistic insights

**Scheme 2:**
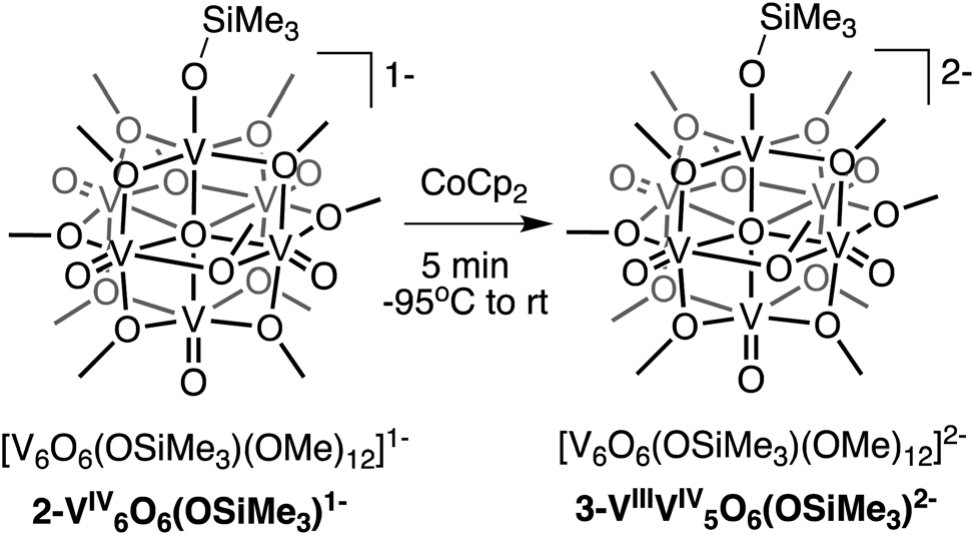
Synthesis of **3-VIIIV5IVO6(OSiMe3)2−**.

Interested in isolating a molecular model for H-doped VO_2_, we next sought to explore the reduction chemistry of complex **2-V6IVO6(OSiMe3)1−**. As described above, the electrochemical profile of **2-V6IVO6(OSiMe3)1−** suggests that binding of the silylium cation to the cluster surface stabilises the reduction of the vanadium oxide assembly. This suggests the possibility of the formation of a reduced siloxide-functionalised POV–alkoxide with an oxidation state distribution of V^III^V^IV^_5_. Chemical reduction of complex **2-V6IVO6(OSiMe3)1−** with CoCp_2_ (*E*_1/2_ = −1.33 V *vs.* Fc^+/0^ in dichloromethane^65^) results in isolation of the reduced assembly, [V_6_O_6_(OSiMe_3_)(OMe)_12_]^2−^ (**3-VIIIV5IVO6(OSiMe3)2−**; Scheme 2), in moderate yield (23%) as confirmed by ^1^H NMR spectroscopy (Fig. S6†) and elemental analysis.

Crystals of **3-VIIIV5IVO6(OSiMe3)2−** suitable for SCXRD were grown from diffusion of diethyl ether into a concentrated solution of the complex in acetonitrile. Broadly, the structure of compound **3-VIIIV5IVO6(OSiMe3)2−** resembles that of **2-V6IVO6(OSiMe3)1−** (Fig. S7,†Table 1, see Table S1† for complete crystallographic data and refinement parameters); the reduced siloxide-functionalised assembly adopts a pseudo-*C*_4v_ point group. Reduction of the cluster core manifests in slight perturbations of the bond metrics of the vanadyl ions of the assembly. In complex **3-VIIIV5IVO6(OSiMe3)2−**, the average V=O_t_ distance of 1.610(4) Å is slightly elongated from that of **2-V6IVO6(OSiMe3)1−** (1.595(6) Å), while the V–O_c_ distances of **3-VIIIV5IVO6(OSiMe3)2−** are shorter than that of **2-V6IVO6(OSiMe3)1−**. These variations in V=O_t_ and V–O_c_ bond distances are inconsistent with structural changes that typically occur across the Lindqvist core following reduction. For example, in the case of the reduction of [V_6_O_7_(OMe)_12_]^−1^ to **1-V6IVO72−**, the V=O_t_ bond distances remain approximately constant (1.60(8) Å *vs.* 1.606(1) Å, respectively), while significant elongation of the V–O_c_ distances are observed (2.297(38) Å *vs.* 2.311(6) Å).^53^

More substantial perturbations of V–O bond distances are observed upon inspection of the bond metrics of the siloxide-functionalised vanadium centre (V1). Significant elongation of the V1–O1 bond of complex **3-VIIIV5IVO6(OSiMe3)2−** (1.929(4) Å) is noted in comparison to that of **2-V6IVO6(OSiMe3)1−** (1.768(3) Å). The V1–O1 bond length is slightly longer than V^III^–OSiR_3_ bond distances reported in the literature (1.852–1.883 Å).^41,66^ This point can be rationalised by discrepancies in coordination geometry of the vanadium centre (pseudo-octahedral V^III^ ion in the case of **3-VIIIV5IVO6(OSiMe3)2−***vs.* pseudo-tetrahedral V^III^ centres in previous work). Taken together, these observations suggest that reduction of the cluster core might be localised to the site-differentiated vanadium centre.

Like **2-V6IVO6(OSiMe3)1−**, complex **3-VIIIV5IVO6(OSiMe3)2−** crystallises with each vanadium centre possessing a unique position within the unit cell. As such, oxidation state distribution assignments of vanadium centres within the cluster core can be determined through BVS calculations (Table S3†). Analysis of the bond metrics for each vanadium centre indicates an oxidation state distribution of V^III^V^IV^_5_; the site-differentiated vanadium centre (V1) possesses V–O bond distances consistent with reduction to V^III^, while the vanadyl ions composing the remainder of the cluster core retain their tetravalent oxidation states. BVS calculations support the hypothesis that reduction of the cluster core is largely localised to the site-differentiated vanadium ion, resulting in the formation of a V^III^–OSiMe_3_ moiety embedded within the Lindqvist assembly. Spectroscopic analysis of H-doped VO_2_*via* XPS suggests that vanadium atoms bound to hydrogenated oxygen atoms also undergo a localised reduction from V^IV^ to V^III^.^8,10,15^

Although comparison of **2-V6IVO6(OSiMe3)1−** and **3-VIIIV5IVO6(OSiMe3)2−** is useful for gaining insight into the perturbations of the assembly upon addition of electron density, a more useful analysis from the perspective of modelling H-atom doping in VO_2_ lies in the comparison of the structural data of **3-VIIIV5IVO6(OSiMe3)2−** to **1-V6IVO72−**. In analogy to spectroscopic data reported for H-doped VO_2_, the general positions of vanadium ions within the Lindqvist lattice do not change substantially upon formation of **3-VIIIV5IVO6(OSiMe3)2−**; V=O_t_ and V–O_c_ lengths of vanadyl ions composing **1-V6IVO72−** and **3-VIIIV5IVO6(OSiMe3)2−** are approximately identical. However, the addition of a hydrogen atom surrogate to a V=O_t_ bond has significant structural ramifications on the site-differentiated vanadium centre. Elongation of the V1–O1 bond is consistent with reduction of the bond order as a result of silyl doping. Analogous atomistic perturbations occur in computational modelling of H-doped VO_2_(R) as a result of decreased charge of the oxido moiety upon hydrogenation.^14^ This distortion ultimately results in reduction of electron correlation due to increased occupancy of V d-levels, suppressing the metal-to-insulator phase transition and promoting metallicity of the bulk material. To date, these subtle structural changes have not been emphasised in discussions of empirical data collected for VO_2_; however, the lattice expansion observed following hydrogen-atom doping indicates that the structural changes supported by *ab initio* calculations, and experimentally modelled in our system, are indeed operative in bulk VO_2_.^14^

Finally, it is useful to discuss the implications of the formation of complex **3-VIIIV5IVO6(OSiMe3)2−** in the context of published reports detailing the mechanism of hydrogen-atom uptake in VO_2_. As described above, the oxidation state distribution of vanadium centres in complex **3-VIIIV5IVO6(OSiMe3)2−** (V^III^V^IV^_5_) reveals the net addition of a H-atom surrogate results in reduction of a site-differentiated metal centre within the vanadium(iv) oxide assembly. This result is analogous to the changes in electronic structure following hydrogen-atom uptake in VO_2_.^8,10,15^ However, the two systems are distinct from one another in their proposed mechanism of H-atom doping. In the case of the formation of the siloxide-functionalised POV–alkoxide cluster, silylium coordination precedes reduction of the assembly. Recall that similar reduction to a POV–alkoxide cluster bearing an oxidation state distribution of V^III^V^IV^_5_ is not possible in the fully oxygenated assembly (*e.g.***1-V6IVO72−**), underscoring the importance of silylium binding to the surface of the assembly prior to injection of electron density.

**Fig. 5:**
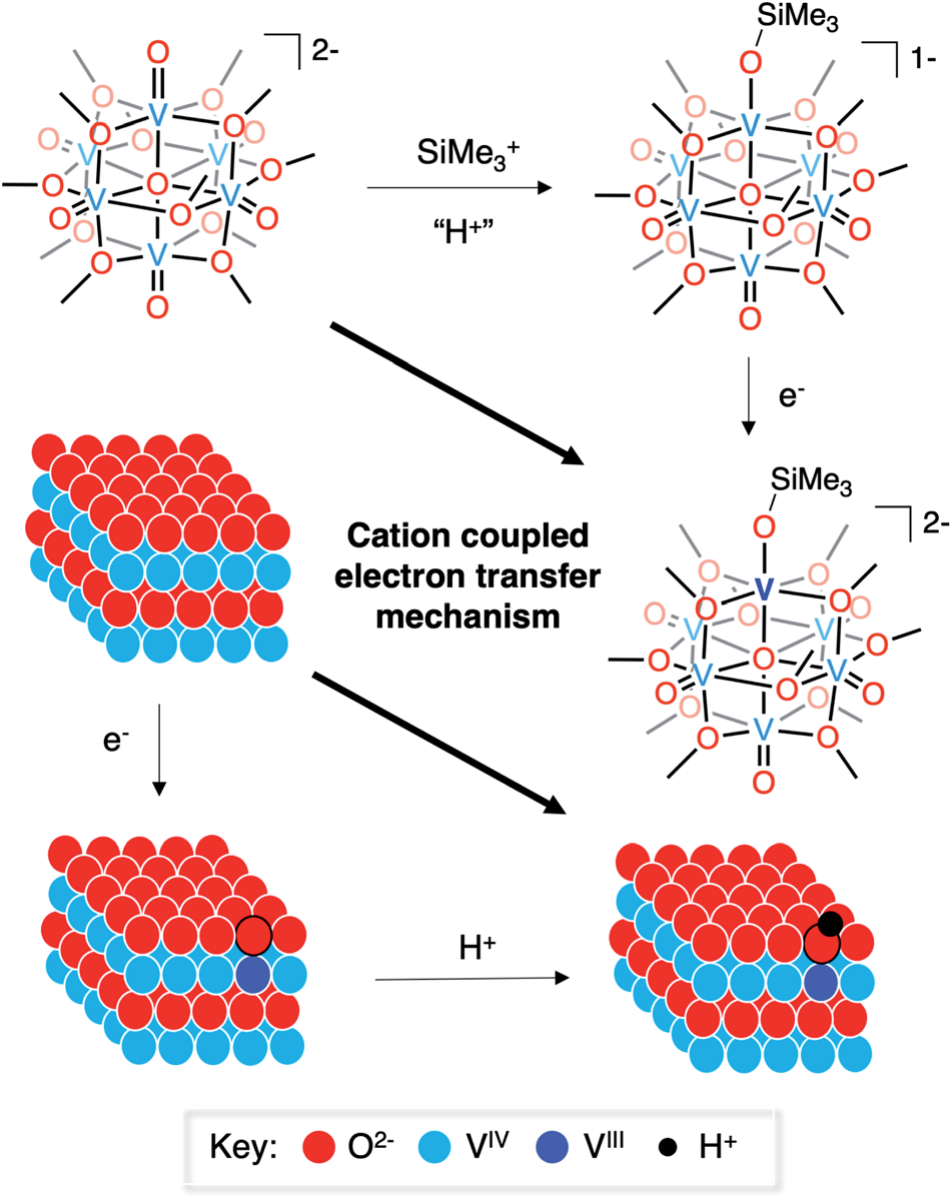
Cation-coupled electron transfer square scheme for the uptake of hydrogen atoms/silyl radicals into vanadium oxide assemblies.

The mechanism of hydrogen-atom addition to complex **1-V6IVO72−** stands in contrast to the suggested mechanism for hydrogen-atom doping in VO_2_ when using acid as the proton source, where injection of electron density is considered the necessary first step to generate H atom dopants in the lattice.^8,15^ The disparity in these two mechanisms, which follow diverging pathways along the thermochemical square schemes (see Fig. 5), suggests the possibility of a third route that could apply to both systems: cation(proton)-coupled electron transfer. It is worth noting that metal oxide materials have been widely reported to perform proton-coupled electron transfer, yielding stable, hydrogenated nanocrystalline scaffolds.^16,67–70^ However, similar mechanisms have not yet been invoked for hydrogen-atom uptake in VO_2_.

## Conclusion

Here, we present the investigation of proton- and hydrogen atom-doping in POV–alkoxide clusters using silylium ions as bulky proton surrogates. This strategy affords isolation of a series of siloxide-functionalised POV–alkoxide clusters, complexes **2-V6IVO6(OSiMe3)1−** and **3-VIIIV5IVO6(OSiMe3)2−**, that serve as models of H-doped VO_2_. The molecular nature of the discrete Lindqvist ion allows for the investigation of atomistic perturbations of V–O bond lengths as a function of silylium coordination. This investigation reveals significant elongation of the V–O bond length following cation binding to the surface of the assembly. Our findings resemble *ab initio* calculations performed for H-doped VO_2_ and provide, for the first time, structural evidence for the formal reduction of the V–O bond order following interstitial doping of the lattice. Silylium coordination stabilises the injection of charge density into the lattice, facilitating the formation of a V^III^ ion at the site-differentiated metal centre. Altogether, these observations qualify the siloxide-functionalised POV–alkoxide cluster as an intriguing model for structural and electronic changes that occur upon hydrogen-uptake in VO_2_.

Additionally, the synthesis and characterisation of the siloxide-functionalised POV–alkoxide clusters present a distinct entry in the atomically precise modelling of dopants using these types of clusters. The site-differentiated vanadium centre that forms upon the surface coordination of a silylium ion appears to remain embedded within the electronic structure of the Lindqvist assembly. This behaviour is in contrast to our previous studies describing the incorporation of substitutional dopants (*e.g.* transition metals, halides) within the hexavanadate core.^24,25,61,62,64,71–73^ In these examples, the heteroatom-functionalised POV–alkoxide clusters adopt electronic structures where the site-differentiated ion is decoupled from the electronic structure of the remaining vanadyl ions of the Lindqvist core. These findings serve to deepen our understanding of the electronic architecture of molecular metal oxides, as well as indicate that the doping motif is relevant to the physicochemical effects observed for a doped material.

Our results suggest alternative mechanisms for electron–proton co-doping of VO_2_. Electrochemical characterisation of the siloxide-functionalised assemblies indicate that silylium coordination is required prior to the reduction of the vanadium oxide assembly. This finding is in contrast to previously reported mechanisms of H-doping in VO_2_(R) that suggest that the injection of electron density precedes the intercalation of protons.^8,15^ While it is possible that the molecular *vs.* extended structure of the VO_2_ assemblies is responsible for the observed differences in mechanism, this study poses important questions regarding the relevance of proton-coupled electron transfer in structurally dynamic materials. Due to the implications of the presented work on the rationalization of the consequences of hydrogen-doping in VO_2_, ongoing investigations in our laboratory are focused on the reactivity of POV–alkoxide clusters with hydrogen atoms and their surrogates, with a goal of more precisely defining the thermochemistry of surface-mediated proton coupled electron transfer (*i.e.* electron–proton co-doping) in VO_2_.

## Experimental

### General considerations

All manipulations were carried out in the absence of water and oxygen using standard Schlenk techniques, or in a UniLab MBraun inert atmosphere drybox under a dinitrogen atmosphere. All glassware was oven dried for a minimum of 4 hours and cooled in an evacuated antechamber prior to use in the drybox. Solvents were dried and deoxygenated on a Glass Contour System (Pure Process Technology, LLC) and stored over activated 3 Å molecular sieves purchased from Fisher Scientific prior to use. Trimethylsilyl trifluoromethylsulfonate (TMSOTf) and cobaltocene (CoCp_2_) were purchased from Sigma Aldrich and used as received. [^*n*^Bu_4_N]_2_[V^IV^_6_O_7_(OMe)_12_] (**1-V6IVO72−**) was prepared according to published procedure.^53^

^1^H NMR spectra were recorded at 500 and 400 MHz on Bruker DPX-500 and Bruker DPX-400 MHz spectrometers locked on the signal of deuterated solvents. All chemical shifts were reported relative to the peak of residual H signal in deuterated solvents. CD_3_CN and CDCl_3_ were purchased from Cambridge Isotope Laboratories, degassed by three freeze–pump–thaw cycles, and stored over fully activated 3 Å molecular sieves. Mass spectrometry analyses were performed on an Advion ExpressionL Compact Mass Spectrometer equipped with an electrospray probe and an ion-trap mass analyser. Direct injection analysis was employed in all cases with a sample solution in acetonitrile.

Single crystals of **2-V6IVO6(OSiMe3)1−** and **3-VIIIV5IVO6(OSiMe3)2−** were mounted on the tip of a thin glass optical fiber (goniometer head) and mounted on a XtaLab Synergy-S Dualflex diffractometer equipped with a HyPix-6000HE HPC area detector for data collection at 100.00(10) K. The structures were solved using SHELXT-2018/2 (ref. 74) and refined using SHELXL-2018/3.^75^ Elemental analyses were performed on a PerkinElmer 2400 Series II Analyzer, at the CENTC Elemental Analysis Facility, University of Rochester.

The XPS measurements of complexes **1-V6IVO72−** and **2-V6IVO6(OSiMe3)1−** were recorded with a Kratos Axis Ultra DLD system equipped with a monochromatic Al Kα (*hν* = 1486.6 eV) radiation. During the measurements, pressure in the main chamber was kept below 1 × 10^−7^ mbar. Charge compensation was carried out *via* a neutraliser with current: 7 × 10^−6^ A, charge balance: 5 eV and filament bias: 1.3 V. The X-ray gun was set to 10 mA emission. Sample preparation was performed under a dinitrogen atmosphere glovebox. The powders were dissolved in dry dichloromethane to obtain a concentrated solution. The solution was drop-casted on cleaned Si wafers, which were grounded to the sample bar by carbon tape. Survey scans from 0 to 1200 eV were carried out to identify the elements present in the sample. Binding energies were referenced to the C 1s peak arising from adventitious carbon with binding energy of 284.8 eV.^24^ The C1s, O1s, Si 2p and V 2p core levels were recorded with a pass energy of 80 eV. 3 scans for vanadium and 2 scans for non-metals were performed. Multiple sample points were used to ensure reproducibility. The V 2p region was increased to 510–540 eV to simultaneously measure the O 1s and V 2p signal in one energy window. XPS data analysis was performed with CasaXPS (Version 2.3.1). The Touggard function was used for background subtraction. The O 1s and V 2p XPS signals were fitted with Gaussian curves. Binding energies were obtained from the same peak fits.

Concentrations of active species for electrochemical analysis (vanadium oxide cluster) and [^*n*^Bu_4_N][PF_6_] used were 1 and 100 mM, respectively, in dichloromethane. Prior to running electrochemical experiments, the supporting electrolyte was recrystallised three times from ethanol and stored under dynamic vacuum. CV measurements were carried out using a Bio-Logic SP 150 potentiostat/galvanostat and the EC-Lab software suite. Glassy carbon disks (3 mm, CH Instruments, USA) were used as working electrodes. Working electrodes were polished using a microcloth pad and 0.05 μm alumina powder. Potentials recorded during CV were measured relative to a nonaqueous Ag/Ag^+^ reference electrode with 10 mM AgNO_3_ and 100 mM [^*n*^Bu_4_N][PF_6_] in acetonitrile (Bio-Logic). A platinum wire served as the counter electrode. All experiments were carried out inside a nitrogen-filled glovebox (MBraun, USA). All CV measurements were IR compensated at 85% with impedance taken at 100 kHz using the ZIR tool included with the EC-Lab software. All redox events were referenced against a ferrocenium/ferrocene (Fc^+/0^) redox couple.

#### Synthesis of [^*n*^Bu_4_N][V^IV^_6_O_6_(OSiMe_3_)(OMe)_12_] (**2-V6IVO6(OSiMe3)1−**)

In a glove box, a 20 mL scintillation vial was charged with **1-V6IVO72−** (0.040 g, 0.030 mmol) and 4 mL acetonitrile. TMSOTf (0.007 g, 0.030 mmol) was dissolved in 2 mL acetonitrile in a separate 20 mL scintillation vial. Both solutions were frozen in a liquid nitrogen cold well. The frozen solutions were taken out of the cold well and TMSOTf was added dropwise with a glass pipette in three portions to the frozen slurry of **1-V6IVO72−** as it thawed. The colour of the solution changed from teal to dark green upon the addition of TMSOTf. The reaction was stirred for an additional 10 minutes after the complete addition of TMSOTf. Residual solvent was subsequently removed under reduced pressure to give a green solid. The crude solid was triturated with small portions of pentane (4 mL) followed by washing with 2 mL of diethyl ether. Dichloromethane was then used to extract the product. After dichloromethane was removed under reduced pressure, the product, **2-V6IVO6(OSiMe3)1−**, was isolated as a dark green solid in good yield (85%, 0.035 g, 0.027 mmol). ^1^H NMR (500 MHz, CD_3_CN): *δ* = 27.50, 24.12, 3.06, 1.57, 1.34, 0.96, 0.76, −9.79 ppm. Elemental analysis for C_31_H_81_NO_19_V_6_Si 0.5 CH_2_Cl_2_ (MW = 1148.17 g mol^−1^) calcd (%): C, 32.95; H, 7.20; N, 1.22. Found (%): C, 32.99; H, 7.21; N 0.89.

#### Synthesis of [^*n*^Bu_4_N][CoCp_2_][V^III^V^IV^_5_O_6_(OSiMe_3_)(OMe)_12_] (**3-VIIIV5IVO6(OSiMe3)2−**)

A 20 mL scintillation vial was charged with complex **2-V6IVO6(OSiMe3)1−** (0.055 g, 0.050 mmol) and 4 mL of dichloromethane. In a separate vial, CoCp_2_ (0.009 g, 0.050 mmol) was dissolved in 2 mL of dichloromethane. Both solutions were frozen in a liquid nitrogen cold well. While thawing, the solution of CoCp_2_ was added in three parts, drop-wise, to the solution of complex **2-V6IVO6(OSiMe3)1−**. Following the complete addition of the reductant, the reaction mixture was stirred for an additional 5 minutes. Solvent was removed under reduced pressure, leaving a dark coloured residue at the bottom of the vial. The product was extracted with cold tetrahydrofuran and filtered through a bed of Celite. After removing tetrahydrofuran under vacuum, the product, complex **3-VIIIV5IVO6(OSiMe3)2−**, was isolated as a blue-green solid in moderate yield (23%, 0.015 g, 0.011 mmol). Crystals suitable for X-ray analysis were grown from vapour diffusion of diethyl ether into a concentrated solution of **3-VIIIV5IVO6(OSiMe3)2−** in acetonitrile; refinement of structural data revealed two tetrabutylammonium ions, suggesting cation exchange during crystallization. ^1^H NMR (500 MHz, CD_3_CN): *δ* = 24.77, 22.75, 3.07, 1.58, 1.33, 0.96, −8.41 ppm. Elemental analysis for C_47_H_117_N_2_O_19_V_6_Si·CH_2_Cl_2_ (MW = 1348.17 g mol^−1^) calcd (%): C, 40.23; H, 8.37; N, 1.95. Found (%): C, 40.47; H, 8.07; N 1.82.

## Data availability

All experimental data supporting the findings in this article have been uploaded as part of the ESI.†

## Author contributions

S. C., E. S., and E. M. M. conceived and planned the experiments. S. C. and E. S. performed the synthesis of complexes and collected all experimental data, except those involving X-ray crystallography and X-ray photoelectron spectroscopy. K. R. S.-L., M. T., and K. E. K. analyzed clusters *via* X-ray photoelectron spectroscopy. W. W. B. collected single crystal X-ray diffraction data and solved the crystal structures. All authors contributed to the writing of the manuscript.

## Conflicts of interest

There are no conflicts to declare.

## Supplementary Material

SC-012-D1SC02809J-s001

SC-012-D1SC02809J-s002

## References

[cit1] Wu C., Feng F., Xie Y. (2013). Chem. Soc. Rev..

[cit2] Wang W., Jiang B., Xiong W., Sun H., Lin Z., Hu L., Tu J., Hou J., Zhu H., Jiao S. (2013). Sci. Rep..

[cit3] Niu C. (2018). Funct. Mater. Lett..

[cit4] Han S., Zou Z., Huo S. (2018). Mater. Sci. Eng..

[cit5] Cui Y., Ke Y., Liu C., Chen Z., Wang N., Zhang L., Zhou Y., Wang S., Gao Y., Long Y. (2018). Joule.

[cit6] Riapanitra A., Asakura Y., Yin S. (2020). Funct. Mater. Lett..

[cit7] Xia C., Lin Z., Zhou Y., Zhao C., Liang H., Rozier P., Wang Z., Alshareef H. N. (2018). Adv. Mater..

[cit8] Chen Y., Wang Z., Chen S., Ren H., Wang L., Zhang G., Lu Y., Jiang J., Zou C., Luo Y. (2018). Nat. Commun..

[cit9] Alivio T. E. G., Sellers D. G., Asayesh-Ardakani H., Braham E. J., Horrocks G. A., Pelcher K. E., Villareal R., Zuin L., Shamberger P. J., Arróyave R., Shahbazian-Yassar R., Banerjee S. (2017). Chem. Mater..

[cit10] Yoon H., Choi M., Lim T.-W., Kwon H., Ihm K., Kim J. K., Choi S.-Y., Son J. (2016). Nat. Mater..

[cit11] Zhang J., He H., Pan B. (2013). Phys. Chem. Chem. Phys..

[cit12] Chenavas J., Joubert J. C., Capponi J. J., Marezio M. (1973). J. Solid State Chem..

[cit13] Wei J., Ji H., Guo W., Nevidomskyy A. H., Natelson D. (2012). Nat. Nanotechnol..

[cit14] Filinchuk Y., Tumanov N. A., Ban V., Ji H., Wei J., Swift M. W., Nevidomskyy A. H., Natelson D. (2014). J. Am. Chem. Soc..

[cit15] Li B., Xie L., Wang Z., Chen S., Ren H., Chen Y., Wang C., Zhang G., Jiang J., Zou C. (2019). Angew. Chem., Int. Ed..

[cit16] Wise C. F., Mayer J. M. (2019). J. Am. Chem. Soc..

[cit17] Pope M. T., Muller A., Polyoxometalate Chemistry: From Topology *via* Self-Assembly to Applications, Berlin, 2001

[cit18] Gumerova N. I., Rompel A. (2018). Nat. Rev. Chem..

[cit19] Lopez X., Carbó J. J., Bo C., Poblet J. M. (2012). Chem. Soc. Rev..

[cit20] Gouzerh P., Proust A. (1998). Chem. Rev..

[cit21] Day V. W., Klemperer W. G., Maltbie D. J. (1987). J. Am. Chem. Soc..

[cit22] Chen Q., Goshorn D. P., Scholes C. P., Tan X. L., Zubieta J. (1992). J. Am. Chem. Soc..

[cit23] Kastner K., Margraf J. T., Clark T., Streb C. (2014). Chem. - Eur. J..

[cit24] Petel B. E., Meyer R. L., Maiola M. L., Brennessel W. W., Müller A. M., Matson E. M. (2020). J. Am. Chem. Soc..

[cit25] Maiola M. L., Petel B. E., Brennessel W. W., Matson E. M. (2020). Dalton Trans..

[cit26] Petel B. E., Matson E. M. (2020). Chem. Commun..

[cit27] Wu C., Hu Z., Wang W., Yang J., Xie Y. (2008). Chem. Commun..

[cit28] Liu L., Cao F., Yao T., Xu Y., Zhou M., Qu B., Pan B., Wu C., Wei S., Xie Y. (2012). New J. Chem..

[cit29] Popuri S. R., Miclau M., Artemenko A., Labrugere C., Villesuzanne A., Pollet M. (2013). Inorg. Chem..

[cit30] Lee S., Ivanov I. N., Keum J. K., Lee H. N. (2016). Sci. Rep..

[cit31] Schreiber E., Petel B. E., Matson E. M. (2020). J. Am. Chem. Soc..

[cit32] Fleming I. (1981). Chem. Soc. Rev..

[cit33] Murata S., Suzuki M., Noyori R. (1980). J. Am. Chem. Soc..

[cit34] Noyori R., Murata S., Suzuki M. (1981). Tetrahedron.

[cit35] Pagano J. K., Dorhout J. M., Waterman R., Czerwinski K. R., Kiplinger J. L. (2015). Chem. Commun..

[cit36] Chu J., Carroll T. G., Wu G., Telser J., Dobrovetsky R., Ménard G. (2018). J. Am. Chem. Soc..

[cit37] Petel B. E., Brennessel W. W., Matson E. M. (2018). J. Am. Chem. Soc..

[cit38] Petel B. E., Fertig A. A., Maiola M. L., Brennessel W. W., Matson E. M. (2019). Inorg. Chem..

[cit39] Petel B. E., Meyer R. L., Brennessel W. W., Matson E. M. (2019). Chem. Sci..

[cit40] Money J. K., Folting K., Huffman J. C., Collison D., Temperley J., Mabbs F. E., Christou G. (1986). Inorg. Chem..

[cit41] Rost M., Görls H., Imhof W., Seidel W., Thiele K. (1998). Z. Anorg. Allg. Chem..

[cit42] Knoth W. H. (1979). J. Am. Chem. Soc..

[cit43] Carlisle Chambers R., Osburn Atkinson E. J., McAdams D., Hayden E. J., Ankeny Brown D. J. (2003). Chem. Commun..

[cit44] Bar-Nahum I., Cohen H., Neumann R. (2003). Inorg. Chem..

[cit45] Mazeaud A., Dromzee Y., Thouvenot R. (2000). Inorg. Chem..

[cit46] Schroden R. C., Blanford C. F., Melde B. J., Johnson B. J. S., Stein A. (2001). Chem. Mater..

[cit47] Mazeaud A., Ammari N., Robert F., Thouvenot R. (1996). Angew. Chem., Int. Ed..

[cit48] Duffort V., Thouvenot R., Afonso C., Izzet G., Proust A. (2009). Chem. Commun..

[cit49] Matt B., Renaudineau S., Chamoreau L. M., Afonso C., Izzet G., Proust A. (2011). J. Org. Chem..

[cit50] Kibler A. J., Martín C., Cameron J. M., Rogalska A., Dupont J., Walsh D. A., Newton G. N. (2019). Eur. J. Org. Chem..

[cit51] Day V. W., Klemperer W. G., Schwartz C. (1987). J. Am. Chem. Soc..

[cit52] Wu C., Feng F., Feng J., Dai J., Peng L., Zhao J., Yang J., Si C., Wu Z., Xie Y. (2011). J. Am. Chem. Soc..

[cit53] Spandl J., Daniel C., Brüdgam I., Hartl H. (2003). Angew. Chem., Int. Ed..

[cit54] Aronica C., Chastanet G., Zueva E., Borshch S. A., Clemente-Juan J. M., Luneau D. (2008). J. Am. Chem. Soc..

[cit55] Mendialdua J., Casanova R., Barbaux Y. (1995). J. Electron Spectrosc. Relat. Phenom..

[cit56] Silversmit G., Depla D., Poelman H., Marin G. B., De Gryse R. (2004). J. Electron Spectrosc. Relat. Phenom..

[cit57] Tian J., Liu F., Shen C., Zhang H., Yang T., Bao L., Wang X., Liu D., Li H., Huang X., Li J., Chen L., Gao H. (2007). J. Mater. Res..

[cit58] O'Connor D. J., Sexton B. A. and Smart R. S. C., Surface analysis methods in materials science, Springer, Berlin ; New York, 2nd edn, 2003

[cit59] Daniel C., Hartl H. (2009). J. Am. Chem. Soc..

[cit60] Daniel C., Hartl H. (2005). J. Am. Chem. Soc..

[cit61] Li F., Carpenter S. H., Higgins R. F., Hitt M. G., Brennessel W. W., Ferrier M. G., Cary S. K., Lezama-Pacheco J. S., Wright J. T., Stein B. W., Shores M. P., Neidig M. L., Kozimor S. A., Matson E. M. (2017). Inorg. Chem..

[cit62] Li F., Meyer R. L., Carpenter S. H., VanGelder L. E., Nichols A. W., Machan C. W., Neidig M. L., Matson E. M. (2018). Chem. Sci..

[cit63] VanGelder L. E., Kosswattaarachchi A. M., Forrestel P. L., Cook T. R., Matson E. M. (2018). Chem. Sci..

[cit64] VanGelder L. E., Matson E. M. (2018). J. Mater. Chem. A.

[cit65] Connelly N. G., Geiger W. E. (1996). Chem. Rev..

[cit66] Veige A. S., Slaughter L. M., Lobkovsky E. B., Wolczanski P. T., Matsunaga N., Decker S. A., Cundari T. R. (2003). Inorg. Chem..

[cit67] Schrauben J. N., Hayoun R., Valdez C. N., Braten M., Fridley L., Mayer J. M. (2012). Science.

[cit68] Valdez C. N., Delley M. F., Mayer J. M. (2018). J. Am. Chem. Soc..

[cit69] Laga S. M., Townsend T. M., O'Connor A. R., Mayer J. M. (2020). Inorg. Chem. Front..

[cit70] Agarwal R. G., Kim H.-J., Mayer J. M. (2021). J. Am. Chem. Soc..

[cit71] Li F., VanGelder L. E., Brennessel W. W., Matson E. M. (2016). Inorg. Chem..

[cit72] Meyer R. L., Brennessel W. W., Matson E. M. (2018). Polyhedron.

[cit73] VanGelder L. E., Brennessel W. W., Matson E. M. (2018). Dalton Trans..

[cit74] Sheldrick G. (2015). Acta Crystallogr., Sect. A: Found. Adv..

[cit75] Sheldrick G. (2015). Acta Crystallogr., Sect. C: Struct. Chem..

