# Supplementary material for: Modelling local structural and electronic consequences of proton and hydrogen-atom uptake in VO2 with polyoxovanadate clusters
Source: Chem Sci. 2021 Aug 25;12(38):12744–53. doi: 10.1039/d1sc02809j (PMC8494032; doi:10.1039/d1sc02809j)
Supplement: SC-012-D1SC02809J-s001 [file SC-012-D1SC02809J-s001.pdf]

Electronic Supplementary Material (ESI) for Chemical Science.
This journal is © The Royal Society of Chemistry 2021

Modelling Local Structural and Electronic Consequences of Proton- and Hydrogen-Atom uptake in VO_2 with Polyoxovanadate Clusters

Sourav Chakraborty, Eric Schreiber, Karla R. Sanchez-Lievanos, Mehrin Tariq, William W. Brennessel, Kathryn E. Knowles, and Ellen M. Matson*

Corresponding author email: matson@chem.rochester.edu

Supporting Information Table of contents.

Fig. S1 ^{1}H-NMR spectra of **1-$V_6O_7^{2-}$** and **2-$V_6O_6(OSiMe_3)^{1-}$**……………………….............S2
Fig. S2 ESI-MS (-ve) spectrum of **2-$V_6O_6(OSiMe_3)^{1-}$** ……………………………………...S2
Table S1 Crystallographic parameters for **2-$V_6O_6(OSiMe_3)^{1-}$** and **3-$V_6O_6(OSiMe_3)^{2-}$**…… S3
Table S2 Bond valence sum calculations for the crystallographically independent vanadium ions in **2-$V_6O_6(OSiMe_3)^{1-}$**………………………………………………………………S3
Fig. S3 X-ray photoelectron spectra of **2-$V_6O_6(OSiMe_3)]^{1-}$** and **$V_6O_7^{1-}$**.................................S4
Fig. S4 ^{1}H-NMR spectra corresponding to stability of **2-$V_6O_6(OSiMe_3)^{1-}$**…………………...S4
Fig. S5 CV of **2-$V_6O_6(OSiMe_3)^{1-}$** with various potential ranges ……………………………....S5
Fig. S6 ^{1}H-NMR spectrum of **3-$V_6O_6(OSiMe_3)^{2-}$**……………………………………..…….S6
Fig. S7 Sigle crystal X-ray structure of **3-$V_6O_6(OSiMe_3)^{2-}$**………………………………….S6
Table S3 Bond valence sum calculations for the crystallographically independent vanadium ions in **3-$V_6O_6(OSiMe_3)^{2-}$**………………………………………………………………S7

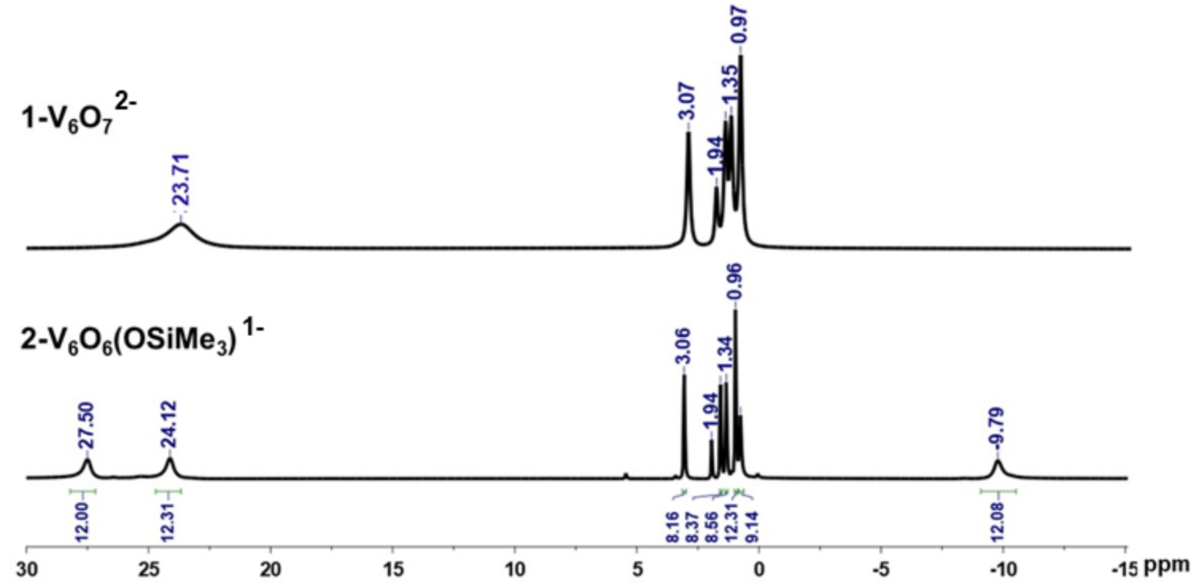

Fig. S1 ^{1}H-NMR spectra of **$1\text{-}V_6O_7^{2-}$** and **$2\text{-}V_6O_6(OSiMe_3)^{1-}$** in CD_3CN, 21ºC.

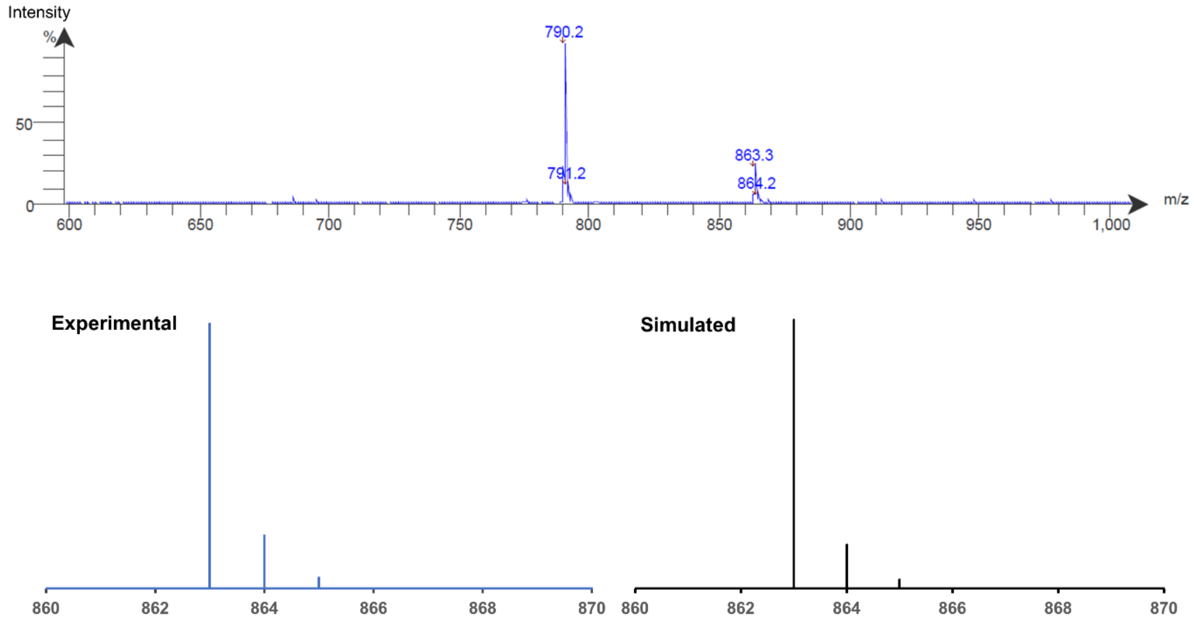

Fig. S2 ESI-MS (-) ve spectrum of **$2\text{-}V_6O_6(OSiMe_3)^{1-}$** in CH_3CN (m/z = 863 amu). The m/z = 790 amu corresponds to **$[V_6O_7(OMe)_{12}]^{1-}$**.

Table S1: Crystallographic parameters of **2-$V_6O_6(OSiMe_3)^{1-}$** and **3-$V_6O_6(OSiMe_3)^{2-}$**.

Name	**2-$V_6O_6(OSiMe_3)^{1-}$**	**3-$V_6O_6(OSiMe_3)^{2-}$**
Empirical formula	$C_{31}H_{81}NO_{19}SiV_6$	$C_{47}H_{117}N_2O_{19}SiV_6$
Formula weight	1105.69	1348.15
Temperature	100.00(10) K	100.00(10) K
Wavelength	1.54184 Å	1.54184 Å
Crystal system	orthorhombic	monoclinic
Space group	P_{bca}	$P2_1/n$
Unit cell dimensions	a = 18.6012(3) Å, α = 90°<br>b = 18.2780(3) Å, β = 90°<br>c = 29.0427(6) Å, γ = 90°	a = 11.77236(17) Å, α = 90°<br>b = 34.8563(7) Å,<br>β = 96.0925(16)°<br>c = 16.6545(3) Å, γ = 90°
Volume	9874.3(3) $Å^3$	6795.4(2) $Å^3$
Z	8	4
Reflections collected	65851	67275
Independent reflections	10356	14212
Goodness-of-fit on F2	1.094	1.060
Final R indices [I>2sigma(I)]	$R1$ = 0.0619, $wR2$ = 0.1762	$R1$ = 0.0840, $wR2$ = 0.2377
CCDC numbers	2058289	2058288

Table S2: Bond valence sum calculations for the crystallographically independent vanadium ions in **2-$V_6O_6(OSiMe_3)^{1-}$** based on X-ray crystallographic data collected at 100 K. Table reflects the results of BVS calculations using V-O bond valence parameters (r_0) for different oxidation states of vanadium.

**2-$V_6O_6(OSiMe_3)^{1-}$**	V1	V2	V3	V4	V5	V6
V(III)	3.788	3.940	4.005	3.944	3.957	3.927
V(IV)	**3.878**	**4.034**	**4.100**	**4.038**	**4.051**	**4.021**
V(V)	4.151	4.300	4.369	4.305	4.319	4.285

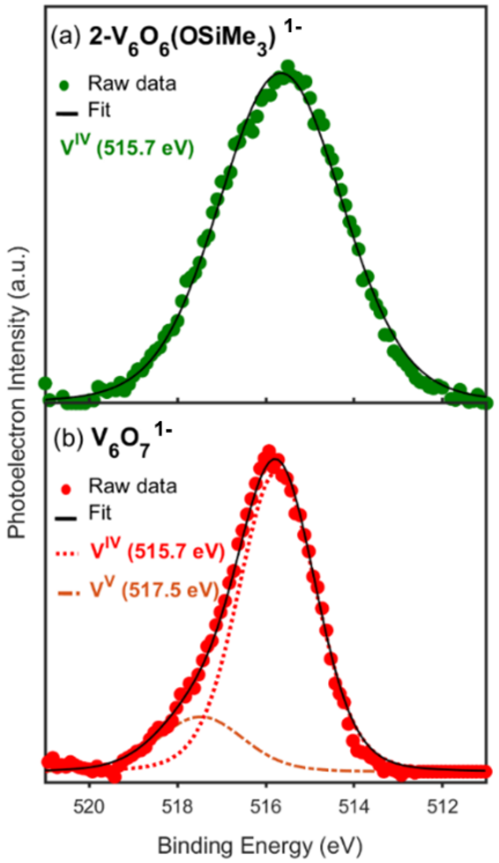

Fig. S3 X-ray photoelectron spectra of the V $2p_{3/2}$ region of (a) **2-$V_6O_6(OSiMe_3)]^{1-}$**, and (b) **$V_6O_7{}^{1-}$**.

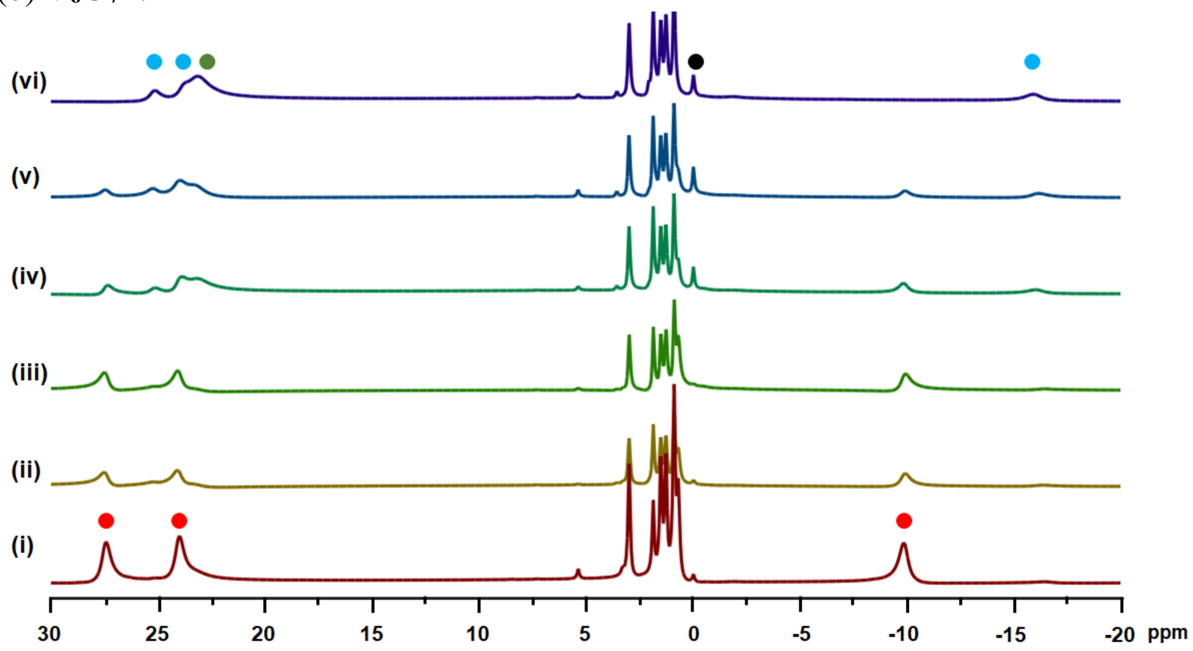

Fig. S4 ^{1}H-NMR spectra of dichloromethane solution of **2-$V_6O_6(OSiMe_3)^{1-}$**. Each spectrum relates to an aliquot of dichloromethane solution of **2-$V_6O_6(OSiMe_3)^{1-}$** removed from the mother liquor after certain time intervals, dried *in vacuo*, and spectrum recorded (in CD_3CN, 21°C) (i) 1hr; (ii) 2hr; (iii) 3hr; (iv) 5 hr; (v) 6hr; (vi) 24 hrs. Red circles correspond to **2-$V_6O_6(OSiMe_3)^{1-}$** (δ = 27.50, 24.12 and -9.79 ppm),

blue circles correspond to **[$V_6O_6(OMe_{12})]^{1-}$** (δ = 25.3, 23.7 and -15.8 ppm),[1] green circle corresponds to **[$V_6O_7(OMe_{12})]^{1-}$** (δ = 23.3 ppm) and black circle corresponds to hexamethyldisiloxane (δ = 0.03 ppm).

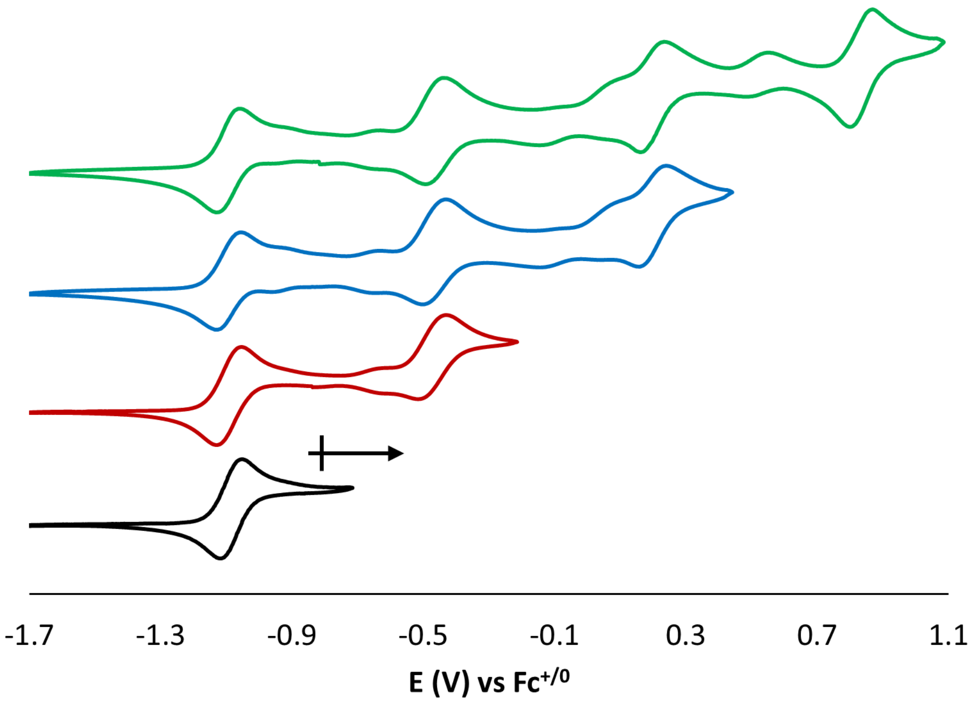

Fig. S5 CV of **2-$V_6O_6(OSiMe_3)^{1-}$** in DCM at 20 mV/s in various potential ranges: -1.7 to -0.72 V (black), -1.7 to -0.22 V (red), -1.7 to 0.4 V (blue), and -1.7 to 1.08 V (green). Oxidative instability is observed, with waves growing in at oxidizing potentials, corresponding with mixtures of **2-$V_6O_6(OSiMe)_3{}^{1-}$**, **[$V_6O_6(OMe_{12})]^{1-}$**, and **[$V_6O_7(OMe_{12})]^{1-}$** $_{12}$.[1, 2]

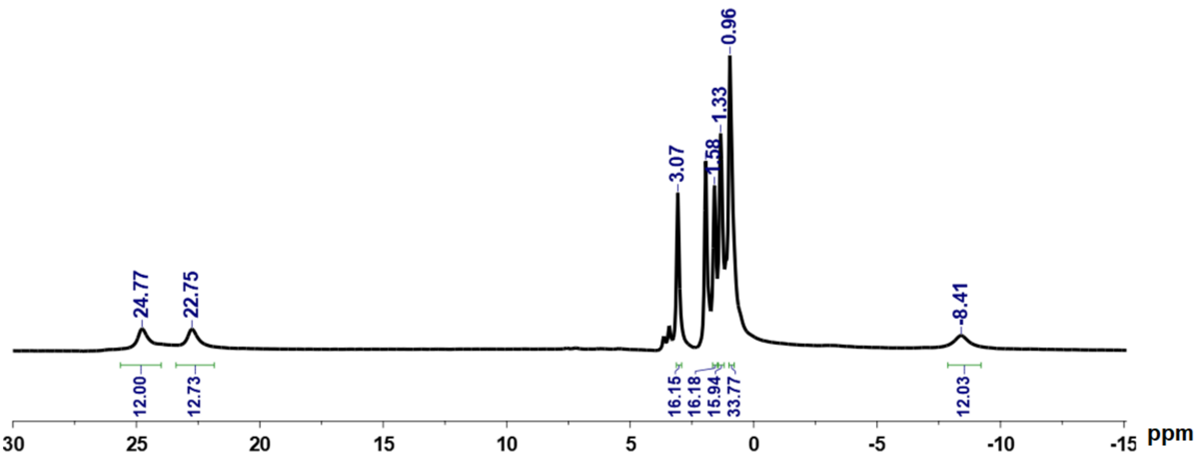

Fig. S6 Molecular structures of **3-$V_6O_6(OSiMe_3)^{2-}$** shown with 30% probability ellipsoids. Hydrogen atoms, counter ions, and solvent molecules have been removed for clarity. Details of the crystallographic parameters are mentioned in Table S1.

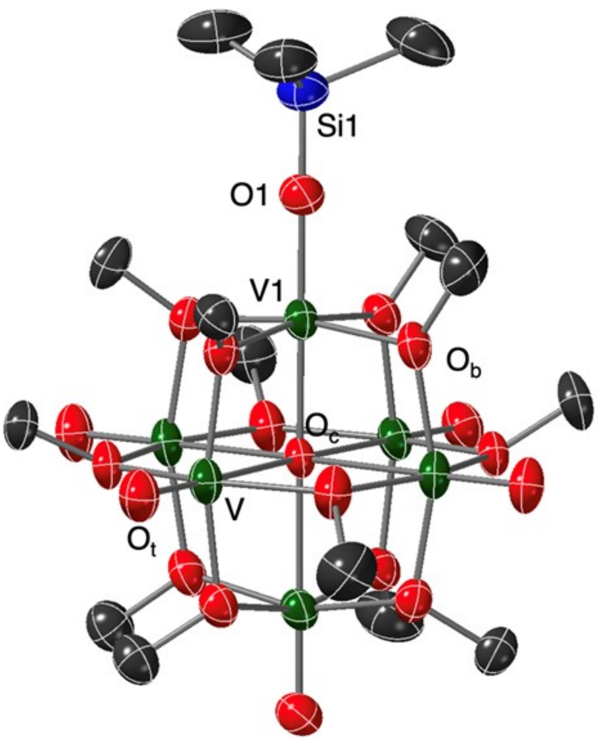

Fig. S7 ^{1}H-NMR spectrum of **3-$V_6O_6(OSiMe_3)^{2-}$** in CD_3CN, 21ºC.

Table S3: Bond valence sum calculations for the crystallographically independent vanadium ions in **3-$V_6O_6(OSiMe_3)^{2-}$** based on X-ray crystallographic data collected at 100 K. Table reflects the results of BVS calculations using V-O bond valence parameters (r_0) for different oxidation states of vanadium.

**3-$V_6O_6(OSiMe_3)^{2-}$**	V1	V2	V3	V4	V5	V6
V(III)	**3.127**	3.933	3.969	3.947	3.913	3.886
V(IV)	3.202	**4.027**	**4.063**	**4.042**	**4.006**	**3.978**
V(V)	3.441	4.295	4.333	4.310	4.272	4.244

References

1. B. E. Petel, W. W. Brennessel and E. M. Matson, *J. Am. Chem. Soc.*, 2018, **140**, 8424-8428.
2. C. Daniel and H. Hartl, *J. Am. Chem. Soc.*, 2005, **127**, 13978-13987.
